# Cognitive impairment in tension-type headache is associated with altered hippocampal functional connectivity

**DOI:** 10.1016/j.isci.2025.113850

**Published:** 2025-10-23

**Authors:** Burak Yulug, Ali Yalcınkaya, Shair Shah Safa, Ayse Karakus, Dila Sayman, Seyda Cankaya, Ceyhun Sayman, Ece Ozdemir Oktem, Behçet Ayyildiz, Sevilay Ayyildiz, Uğur Aylak, Bernis Sutcubası, Ramazan Karaca, Mehmet Ozansoy, Umutcan Duran, Halil Aziz Velioglu, Lutfu Hanoglu, Adil Mardinoglu

**Affiliations:** 1 Alanya Alaaddin Keykubat University, Department of Neurology, Antalya, Turkey; 2 Functional Imaging and Cognitive-Affective Neuroscience Lab (fINCAN), Health Sciences and Technology Research Institute (SABITA), Istanbul Medipol University, Department of Neurology and Neuroscience, Istanbul, Turkey; 3 Anatomy PhD Program, Graduate School of Health Sciences, Kocaeli University, Istanbul, Turkey; 4 Technical University of Munich, School of Medicine, Department of Neuroradiology, Munich, Germany; 5 Technical University of Munich, School of Medicine, TUM-NIC Neuroimaging Center, Munich, Germany; 6 Department of Neuroscience, Bahcesehir University, Ankara, Turkey; 7 Department of Psychology, Faculty of Humanities and Social Sciences, Acibadem University, Istanbul, Turkey; 8 Center for Psychiatric Neuroscience, Feinstein Institute for Medical Research, Manhasset, New York, NY 11030, USA; 9 Science for Life Laboratory, KTH-Royal Institute of Technology, 17165 Stockholm, Sweden

**Keywords:** Clinical neuroscience, Cognitive neuroscience

## Abstract

Tension-type headache (TTH) is a widespread primary headache disorder that causes mild to moderate pain, which may be seen together with cognitive deficits. It is unclear if TTH-linked cognitive impairment is associated with functional alterations. Seventy-five participants were enrolled in the study. Mini Mental State Evaluation (MMSE) and Montreal Cognition Assessment (MoCA) tests were applied to evaluate cognitive impairment. A neuroimaging analysis was applied to determine whether the hippocampus responsible for pain and cognition was affected in TTH patients. Our functional data revealed significant alterations in the connectivity of the subiculum, hippocampal fissure, and left whole hippocampus. Among the significant functional brain alterations observed, the right subiculum consistently interacted with MoCA scores and increased pain intensity. Our findings suggest that TTH patients with cognitive impairment may exhibit unique functional alterations in the hippocampus. This suggests a potential negative association between pain modulation and cognitive processes in the hippocampus that may be responsible for the increased risk of dementia in these patients.

## Highlights

• The right subiculum, middle temporal, and frontal gyrus process both pain and cognition
• Hippocampal and middle temporal regions relate to cognitive decline in TTH pathology
• MoCA scores and pain intensity are negatively correlated in the TTH group

## Graphical abstract

**Figure:**
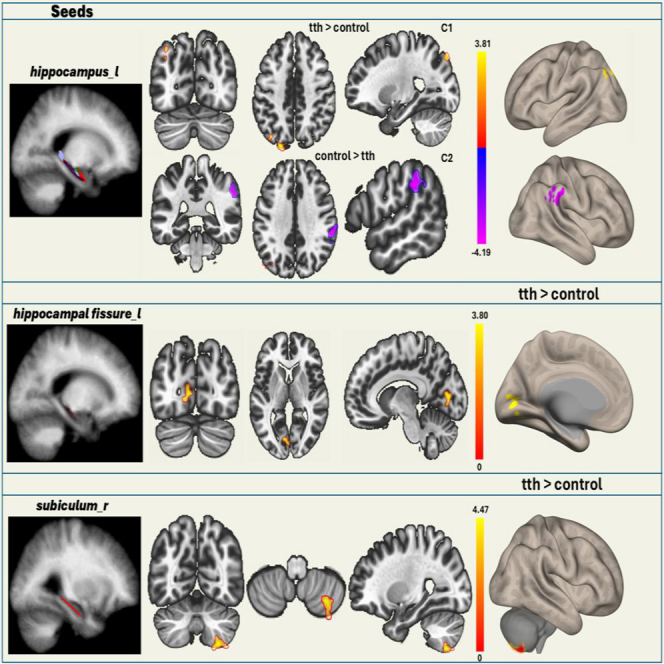

## Introduction

Tension-type headache (TTH) is a ubiquitous primary headache disorder characterized by the occurrence of mild to moderate headache episodes typically manifesting as bilateral, pressing, or tightening sensations.^1^ Individuals with TTH frequently experience additional health issues, including fibromyalgia, temporomandibular joint dysfunction, sleep disturbances, depression, and anxiety.^2^^,^^3^^,^^4^^,^^5^^,^^6^ It is important to note that individuals diagnosed with TTH not only face the direct health impacts described earlier^7^ but also often struggle with cognitive deficits.^8^^,^^9^^,^^10^ However, in contrast to the substantial body of research into migraine-related cognitive and mood impairment,^11^^,^^12^ there is a notable scarcity of studies focused on these aspects in patients with TTH.^13^^,^^14^ Moreover, the limited research that does exist has produced inconsistent results. For instance, Waldie et al. found no cognitive performance difference between adult TTH patients and healthy individuals, suggesting that any cognitive issues in childhood TTH may stem from diminished educational opportunities due to the social and health impacts of the condition.^13^ Further investigations into the link between TTH and the risk of dementia have been inconclusive, partly due to the younger ages of study participants, limiting the applicability of the findings to the broader dementia population.^15^^,^^16^ Other studies have indicated that TTH patients may experience impairments in visuospatial skills, executive functions, and attention.^11^^,^^14^^,^^17^ However, these have not adequately considered how depression, anxiety, and insomnia might affect cognitive outcomes, making firm conclusions elusive. Collectively, these studies underscore the potential capacity of advanced neuroimaging methods to uncover the underlying brain mechanisms responsible for TTH-associated cognitive problems.^18^ From that perspective, several TTH studies^19^^,^^20^ have reported abnormal brain function and altered gray matter volume and white matter integrity,^9^ with some studies specifically identifying the hippocampus as a region of interest.^21^ However, a specific relationship between hippocampal activity and impaired cognition has not yet been shown in TTH. Regrettably, this also holds true for the functional data. While robust evidence exists for the role of regional homogeneity (ReHo) and amplitude of low-frequency fluctuations (ALFFs) in pain processing in TTH,^22^^,^^23^ results regarding alterations in hippocampal regions are scarce.^22^^,^^23^^,^^24^^,^^25^

The aforementioned discrepancy may stem from the fact that the regions identified as significant in these studies could be indicative of altered central pain processing, rather than cognition. This, in turn, makes it difficult to establish a clear differentiation between brain regions involved in cognition and pain processing. Another constraint of previous studies is the confounding effect of mood on headache. This necessitates further investigations into the mechanisms of mood in TTH in order to eliminate the potential effect of psychological distress on headache and cognition by evaluating strategic regions, such as the hippocampus, which plays a multifaceted role in mediating cognition, mood, and pain processing.^26^^,^^27^ This is suggested with previous studies emphasizing the role of hippocampal subregions in neurological and psychiatric diseases^28^ associated with cognitive impairment, such as Alzheimer’s disease (AD),^29^ and major depressive disorder (MDD).^30^ These findings are suggested with the fact that, reduced volume, decreased neurogenesis, and altered neuroplasticity in the hippocampal regions are among the morphological and functional alterations linked to depression and chronic pain mediated by a decrease in neurotrophic factors and an increase in pro-inflammatory factors.^31^

In light of all these gaps, a more focused approach that examines functional hippocampal alterations with appropriate adjustments is now needed to accurately delineate the cognition-related changes associated with TTH.

The study primarily aims to determine whether the functional hippocampal changes observed in TTH reflect direct cognitive impairments or are simply a byproduct of altered central pain processing and mood disturbances. Essentially, the research addresses the gap in our understanding of how TTH might lead to cognitive deficits by using advanced neuroimaging methods to clearly differentiate between regions involved in pain processing and those directly related to cognitive function. So far as we are aware, no previous integrated study has evaluated the neural correlations of a possible link between cognition and pain in TTH patients in such depth manner. Here, we conducted neuropsychological tests and seed-based resting state functional connectivity (rsFC) analyses on age-, sex- and educational years-matched healthy controls (HCs) and patients with TTH. The bilateral hippocampi were chosen as seed regions because of their important roles in cognition.

## Results

### Demographic features and clinical test scores

The participants’ demographic features and clinical test scores are summarized in Table 1. No significant differences were observed in terms of age (*p* = 0.926) and years of education (*p* = 0.088). However, we observed a significant difference in Montreal Cognition Assessment (MoCA) scores between TTH and control groups (Mann-Whitney U Test, *p* = 0.004, Table 1). MoCA scores were significantly lower in the TTH patients than in the control group (Table 1). We also found significant difference between groups in terms of Hamilton Depression Rating Scale (HDRS) scores (*p* < 0.001, Mann-Whitney U test, Table 1). There were no differences in terms of cognitive scores between acute and chronic type of TTH after adjusting for different HDRS and Hamilton Anxiety Scale (HAS) scores observed between the two groups (analysis of covariance, *p* > 0.05). The pain intensity of TTH patients (mean ± SD) was 4.34 ± 2.04, and the pain frequency (mean ± SD, days per month) was 16.6 ± 9.12.

**Table 1:** Demographic features and clinical test scores of the two groups are shown in the table

	Tension-type headache *n* = 29		Control *n* = 46		*p* value
	Mean ± SD	Median (IQR)	Mean ± SD	Median (IQR)	
Years of education	10.96 ± 4.24	12.00 (8)	12.76 ± 4.37	12.00 (4)	0.088
Age	36.51 ± 13.05	33.00 (23)	39.58 ± 18.13	32.00 (30)	0.926
MoCA	24.27 ± 2.31	24.00 (3)	26.13 ± 2.51	26.00 (4)	0.004^a^
MMSE	29.51 ± 0.73	30 (1)	29.28 ± 0.93	30 (1)	0.339
HDRS	9.20 ± 4.92	10 (8)	5.58 ± 3.65	6 (4.75)	<0.001^a^
HAS	14.10 ± 8.86	13 (13)	12.62 ± 8.17	11 (12)	0.527
Trail making part B	0.83 ± 0.39	1 (0)	0.87 ± 0.34	1 (0)	0.625
Visuospatial skills	3.62 ± 0.68	4 (1)	4.17 ± 0.68	4 (1)	<0.001^a^
Naming	2.58 ± 0.63	3 (1)	2.80 ± 0.40	3 (0)	0.120
Attention	5.51 ± 0.69	6 (1)	5.80 ± 0.45	6 (0)	0.025^a^
Sentence repetition	0.90 ± 0.90	1 (2)	1.39 ± 0.78	2 (1)	0.018^a^
Fluency	0.79 ± 0.41	1 (0)	0.94 ± 0.25	1 (0)	0.069
Abstraction	1.24 ± 0.74	1 (1)	1.61 ± 0.61	1 (2)	0.022^a^
Recall	2.76 ± 1.40	3 (2)	2.89 ± 1.54	3 (2)	0.676
Orientation	5.93 ± 0.26	6 (0)	5.96 ± 0.21	6 (0)	0.645
Gender (female *n*, %)	21 (%72)		25 (%54)		0.122

a There were no significant differences in terms of age and education year between the two groups. On the other hand, MoCA and HDRS scores showed significant difference (*p* = 0.004, Mann-Whitney U test was performed.) Also, MoCA subtests visuospatial skills, attention, sentence repetition, and abstraction showed significant difference between groups. (Abbreviations: MoCA, Montreal Cognition Assessment; MMSE, Mini Mental State Evaluation; HDRS, Hamilton Depression Rating Scale; HAS, Hamilton Anxiety Scale).

### Functional connectivity results

#### Functional connectivity group differences

**Figure 1:**
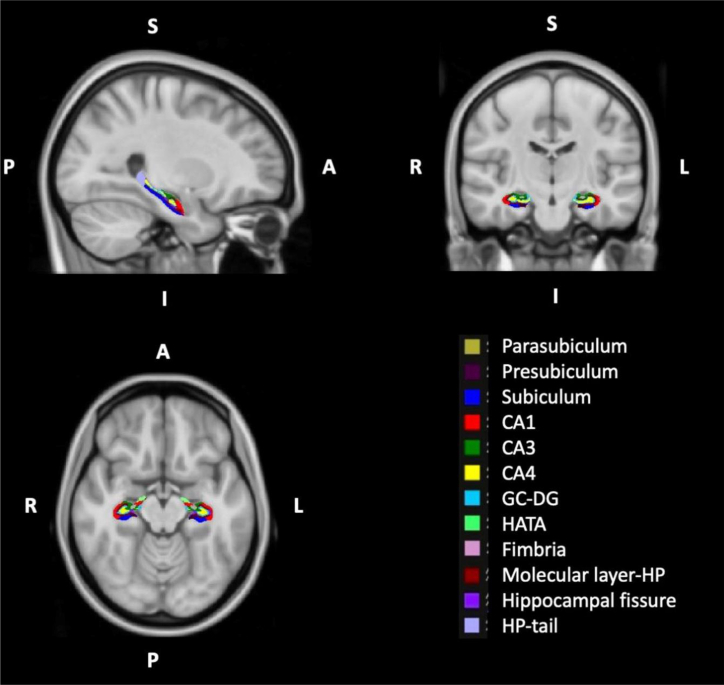
The bilateral hippocampal subfield seeds

**Figure 2:**
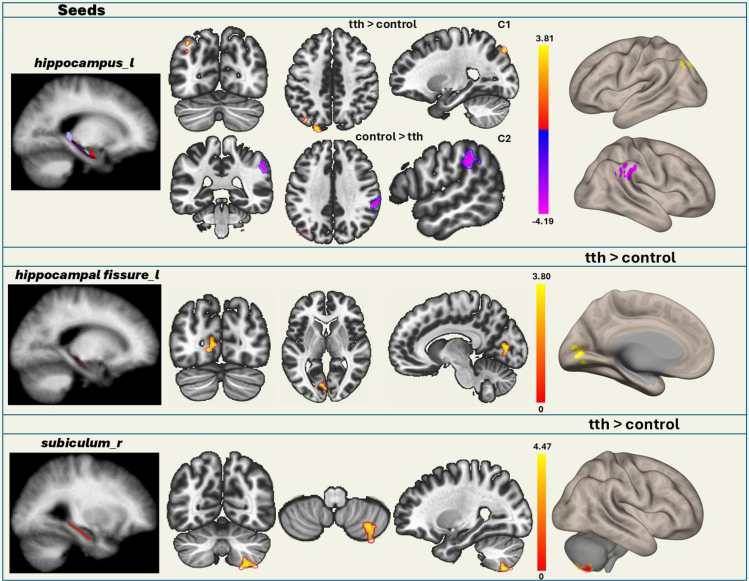
Functional connectivity differences of hippocampal subfields between the two groups The connectivity of the left whole hippocampus with the right supramarginal gyrus and right parietal operculum cortex decreased, while the connectivity with the left superior lateral occipital cortex and left occipital pole significantly increased in the TTH group. The left hippocampal fissure in the TTH group showed significantly increased functional connectivity with the left lingual gyrus and intracalcarine cortex. The right subiculum body in the TTH group showed significantly increased functional connectivity with the right cerebellum in comparison to healthy controls. The regions depicted on brain maps are described in Table 2.

**Figure 3:**
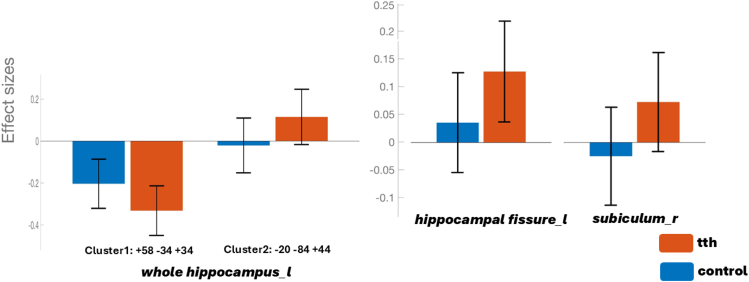
Effect size of hippocampal subregions showing functional connectivity differences between patients with TTH and control groups The effect sizes (Fisher z-transformed correlation coefficients) are shown for two groups: the blue bars represent the control group, and the red bars represent the TTH group. The effect size reflects the strength of functional connectivity in ROIs, which shows significant connectivity differences between the two groups. The coordinates on the *x* axis correspond to the peak MNI coordinates (from Table 2), related to the ROIs that exhibited functional connectivity differences between the two groups.

In the analysis of functional connectivity of the hippocampus and its subfields (Figure 1) with other brain regions, the left hippocampal fissure in the TTH group showed significantly increased functional connectivity with the left lingual gyrus and left intracalcarine cortex (Figures 2 and 3; Table 2). Similarly, the right subiculum body in the TTH group showed significantly increased functional connectivity with the right cerebellum in comparison to healthy controls. Also, the left whole hippocampal connectivity showed both increased and decreased functional connectivity in the TTH group. Herein, the left superior lateral occipital cortex and left occipital pole were significantly increased whereas the right supramarginal gyrus and right parietal operculum cortex were decreased in the TTH group (Figures 2 and 3; Table 2).

**Table 2:** Functional connectivity differences between the two groups

Seeds	Cluster locations	Cluster coordinates	Size	t-max	*p* value
**TTH > Control**					

Left hippocampus	left superior lateral occipital cortex, left occipital pole	−20−84 + 44	139	4.93	0.023516
Left hippocampal fissure	left lingual gyrus, left intracalcarine cortex	−12−74 -02	114	4.85	0.036351
Right subiculum body	right cerebellum	+26–64−58	120	5.25	0.024624
Left hippocampus	right supramarginal gyrus, right parietal operculum cortex	+58−34+34	149	−5.18	0.016247

The table shows the seeds exhibiting functional connectivity differences between the TTH group and the control group. The left hippocampal fissure and right subiculum show increased connectivity in the TTH group. Additionally, the left whole hippocampus shows both increased and decreased connectivity in the TTH group. The t-values reported in the table correspond to the *post hoc* comparisons between the groups. The *p* value reflects differences between groups, assessed using one-way analyses of covariance (ANCOVA), with control variables taken into account. (Voxel-wise significance was set at *p* < 0.001, and cluster-level significance was determined with *p* FWE < 0.05.).

#### Functional connectivity regression analysis

**Figure 4:**
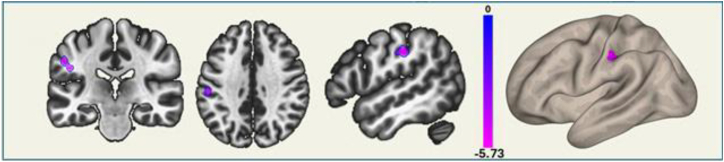
Functional connectivity in the TTH group, analyzed based on the interaction between MoCA scores and pain intensity The connectivity between the *right subiculum* and left postcentral gyrus and left supramarginal gyrus correlates negatively with pain intensity depending on MoCA scores. The regions depicted on brain map are described in Table 3.

**Figure 5:**
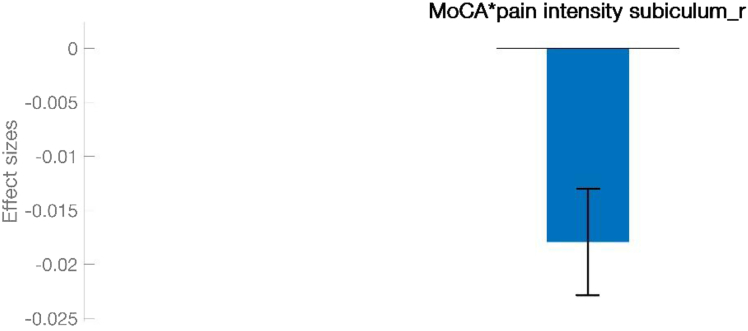
Effect size of ROI showing significant functional connectivity in the TTH group based on the interaction between MoCA scores and pain intensity The effect size reflects the strength of functional connectivity in ROI that shows significant connectivity differences based on the pain intensity ∗ MoCA interaction in the TTH group.

Among the functionally significant regions, the right subiculum’s connectivity with the left postcentral gyrus and left supramarginal gyrus was significantly interacted with the MoCA scores. (Figures 4 and 5; Table 3).

**Table 3:** Functional connectivity in the TTH group, analyzed based on the interaction between MoCA scores and pain intensity

Seeds	Cluster locations	Cluster coordinates	Size	t-max	*p* value	R
**Negative correlation with pain intensity**						

Right subiculum	left postcentral gyrus, left supramarginal gyrus	−46−24+30	60	−6.28	0.039898	−0.442

Specifically, the connectivity between the right subiculum and the left postcentral gyrus, along with the left supramarginal gyrus, negatively correlates with pain intensity depending on MoCA scores.

## Discussion

In this study, we found that, compared with healthy controls, patients with TTH exhibited impaired cognition and increased anxiety, which showed lower MoCA scores and higher HAS and HDRS scores. The rsFC features of left hippocampus and hippocampal subfields with the left occipital cortex, left lingual gyrus, and right cerebellum were significantly different in TTH patients compared to HCs. Moreover, the rsFC strength of the subiculum with the right cerebellum was associated with impaired cognition and increased headache frequency in TTH patients. Although participants with TTH exhibited significantly reduced cognitive performance compared to the healthy controls, no significant difference was observed in terms of cognitive scores between acute and chronic types of headache patients.

Although our findings contradict recent findings by Wang et al., showing no significant differences in hippocampal connectivity between TTH and healthy controls, it suggests previous structural studies indicating the role of hippocampus in adult patients with high-frequency TTH.^32^ In the light of the existing literature, which highlights the adverse impact of acute pain on cognitive function,^33^ our findings of functional alterations in relevant brain regions critical to cognition (such as, left hippocampus, hippocampal fissure, and subiculum) support the hypothesis that the processing of acute episodes of pain may interact negatively with cognitive functions, potentially leading to a decline in cognitive capacities. Our finding of significantly different hippocampal connectivity changes in group comparisons is also supportive of recent data concerning an increased risk of dementia in patients with TTH.^8^^,^^9^^,^^10^ Within this framework, the current results align with recent studies highlighting the undeniable role of the hippocampus in AD as well as during the experience of pain.^34^^,^^35^ An additional example supporting our findings is a recent study reporting that the enlargement of the hippocampal fissure was a prominent finding associated with medial temporal lobe atrophy in patients with AD compared to healthy individuals.^36^ Collectively, these results are consistent with our own observations of altered hippocampal connectivity in TTH patients with cognitive impairment, which corresponds with its known role in predicting the development of AD, particularly in early stages such as mild cognitive impairment.^37^

Among the aforementioned hippocampal regions identified as differing functionally significant between the TTH patients and healthy controls, the right subiculum emerged as a unique region that consistently interacted with the processing of cognition and pain. From that perspective, our present study proposes that episodes of pain, particularly in terms of intensity, may lead to increased activation in brain regions associated with the encoding of painful memories at the expense of cognitive functioning.^38^ This aligns with several human and animal studies,^26^^,^^27^^,^^39^ suggesting a significant association between reduced hippocampal volume and memory impairments in the presence of chronic pain.^40^^,^^41^ A good clinical example of this could be recent interesting data indicating that even acute pain may impair executive functions.^33^^,^^42^ This is consistent with our results showing a significant negative correlation between MoCA and pain intensity scores that uniquely interacted on the right subiculum body’s connection with the left postcentral gyrus and left supramarginal gyrus (Figures 4 and 5; Table 3).

It was, therefore, not unreasonable to assume that this cognitively relevant region might also play a mediating role in pain and cognitive impairment, a finding worthy of further discussion. For instance, O’Mara^43^ confirmed the role of the subiculum in spatial information processing, memory, stress responses, and motivated behavior, which are all relevant to various neurocognitive processes. This aligns well with a novel study by Gao et al. suggesting that short-term intrinsic plasticity can modulate pain-related neuronal circuits through subiculum’s unique sensitizing function on the retrosplenial cortex.^44^ In addition, clinically relevant data reported by Chen et al. in patients with medication-overuse headaches showed a negative correlation between headache characteristics, anxiety scores, and right subiculum activity.^25^ This implies that the right subiculum significantly influences the pathophysiology of such headaches. Confirming the role of the subiculum in pain, another study reported that trigeminal neuralgia was associated with decreased right subiculum activity compared to a control group.^45^

Although we specified the presence of depression as an exclusion criterion, we observed higher subclinical HDRS scores in the TTH patients than in the control group, which might represent a possible confounding factor affecting our results. In order to eliminate the impact of subtle depressive symptoms on cognition, we therefore adjusted HDRS scores in group difference functional connectivity analysis.

In conclusion, our present findings suggest that TTH patients with cognitive impairment may exhibit unique functional alterations in brain regions, as confirmed with functional analyses. This suggests a potential negative association between pain modulation and cognitive processes in these regions that may be responsible for the increased risk of dementia in these patients and may pave the way for new therapeutic strategies to address cognitive deficits in TTH patients.

### Limitations of the study

Limitations of this study also include its cross-sectional nature, which makes it difficult to infer causal and temporal relationships. Furthermore, functional and structural MRI acquisitions were limited in spatial resolution, and low-quality signal effects may have biased results. To minimize potential confounding by signal heterogeneity in our analyses, we applied a quality check of the regions of interest. Finally, due to the cross-sectional design of our study, we cannot draw conclusions regarding the order of pathological events driving subregional brain changes, which should be addressed in ongoing longitudinal studies. It is also important to mention that the findings of this study should be interpreted cautiously and irrespective of direction of connectivity, especially in terms of identifying brain regions that are likely to contribute to cognitive impairment, especially in pain conditions such TTH.^46^

## Resource availability

### Lead contact

Further information and requests for resources and reagents should be directed to and will be fulfilled by the lead contact, Burak Yulug (burak.yulug@alanya.edu.tr).

### Materials availability

This study did not generate new unique reagents.

### Data and code availability

• Data: Data are available on request due to privacy/ethical restrictions. Further information for data should be directed to and will be fulfilled by the lead contact, Burak Yulug (burak.yulug@alanya.edu.tr).
• Code: This study did not use any custom or publicly available code.

## Acknowledgments

All authors would like to thank all participants who took part in this study. No funding support was received from any institution for this work.

## Author contributions

Conceptualization: B.Y. and S.S.S. Formal analysis: U.D., H.A.V., L.H., and A.M. Investigation: B.Y., H.A.V., S.C., and A.K. Methodology: B.Y. and S.S.S. Project administration: B.Y., S.S.S., B.S., and U.A. Software: B.A., S.A., and A.Y. Supervision: B.Y., S.S.S., H.A.V., L.H., and A.M. Validation: S.C., R.K., C.S., and M.O. Visualization: C.S., D.S., E.O.O., and M.O. Writing – original draft: B.Y., S.S.S., S.C., and A.K. Writing – review and editing: B.Y., H.A.V., L.H., A.M., and S.C.

## Declaration of interests

The authors declare no competing interests.

## STAR★Methods

### Key resources table

**Table:** 

REAGENT or RESOURCE	SOURCE	IDENTIFIER
**Software and algorithms**		

MRIcroGL	https://www.nitrc.org/projects/mricrogl	Version 1.2
CONN functional connectivity toolbox	https://web.conn-toolbox.org	Version 21a
MATLAB	MathWorks	R2023a
SPM12 toolbox	https://www.fil.ion.ucl.ac.uk/spm/software/spm12	–
ART (Artifact Detection Tool)	Included in CONN toolbox	–
FreeSurfer	https://surfer.nmr.mgh.harvard.edu	Version 7.1.1
Statistical analysis (ANOVA, GLM)	Conducted with CONN and MATLAB	–
G∗Power	https://www.psychologie.hhu.de/arbeitsgruppen/allgemeine-psychologie-und-arbeitspsychologie/gpower	Version 3.1
Jamovi	https://www.jamovi.org/	Version 2.3.19.0

### Experimental model and study participant details

Seventy-five (29 TTH patients (14 episodic and 15 chronic) and 46 healthy controls) participants were enrolled in the study. 21 (%72) participants of the TTH group and 25 (%54) participants of the control group were female. The participants were recruited from the Alanya Alaaddin Keykubat University, Department of Neurology and Clinical Neurosciences, Antalya/Turkey. Participants with neurodegenerative, neuropsychiatric chronic metabolic disease, or previous histories of trauma and any other headache types were excluded from the study. The tension-type headache diagnosis was done by an experienced neurologist based on International Headache Committee diagnostic criteria. All evaluations of the control group were performed during the study, and the national general health system was employed to verify that none of the control group members had ever been diagnosed with tension-type headache or any other headache type, such as migraine. The Mini-Mental State Examination (MMSE) and Montreal Cognitive Assessment (MoCA) were applied to evaluate the cognitive impairment of the participants, while the MMSE test was used to rule out dementia. All subjects with tension-type headaches were enrolled during the interictal period. Also, the Hamilton Depression Rating Scale (HDRS) was used to assess depression and Hamilton Anxiety Scale (HAS) was used to assess anxiety in both the TTH and control groups, and participants who were using antidepressant or anti-anxiety medications at the time of evaluation were excluded from the study. Participants were diagnosed with TTH based on the International Classification of Headache Disorders (ICHD-3) criteria. Patients' pain severity was assessed using VAS (Visual Analog Scale) scores and rated it on a scale from 0 to 10. Approval of the study was granted by the Istanbul Medipol University ethical committee (Ethical report number 16072023/289). G∗power (ver. 3.1.6.6) software was used to determine the sample size. This showed that a sample size of 16 subjects would be required for 90% power with a significance level of α=0.05.^47^

### Consent statement

All participants provided and signed informed consent.

### Method details

#### MRI data acquisition

Structural and resting-state fMRI were conducted using a Signa Explorer MR device (General Electric Company, USA) at Alanya Alaaddin Keykubat University. Each T1-weighted structural scan consisted of 190 slices (TR/TE: 8.1/3.7), FOV 256 x 256 x 190 mm (FHxAPxRL), and a voxel size of 1 x 1 x 1 mm. Echo-planar imaging sequences (EPI) were used to record fMRI scans in the resting state. The scanning procedure had a duration of approximately 12 minutes and 300 volumes were recorded with the following parameters: TR 2230 ms, TE 30 ms, FOV 240 x 240 x 140 mm (RLxAPxFH), voxel size 3 x 3 x 4 mm, flip angle 77°, and slice number 35. All participants were instructed to close their eyes, remain still and relaxed, clear their minds, and resist falling asleep before the scan.

#### Extraction of hippocampal seeds

The bilateral hippocampal subfield seeds were obtained using FreeSurfer software (version 7.1.1). The “recon-all” pipeline was utilized to segment the MNI 1-mm standard space. Following this segmentation, the hippocampus segmentation tool in FreeSurfer was used to delineate the hippocampal subfields. In the structural analysis of the hippocampus subfields, the hippocampal fissure and subiculum, which were different between the groups, were selected as seeds (Table S1). The seeds, initially in FreeSurfer’s (.mgh/.mgz) format, were converted to Nifti (.nii/.nii.gz) format and subsequently incorporated into the CONN toolbox to enable seed-based connectivity analyses (Figure 1).

#### Analysis of rsFC

The participants’ structural and resting-state functional images were converted from DICOM to NIfTI format using MRIcroGL (https://www.nitrc.org/projects/mricrogl) and then imported into the Functional Connectivity Toolbox (CONN v21a, https://web.conn-toolbox.org), a Matlab- and SPM-based software (https://www.fil.ion.ucl.ac.uk/spm/software/spm12/). The images underwent preprocessing using the default functional and anatomical pipeline, which includes realignment & unwarp, slice-timing correction, outlier detection, segmentation, normalization, and smoothing. Head movement realignment was set at the 97th percentile with linear motion parameters >0.9 mm and global signal z >3 thresholds. Outlier functional images were detected using the artefact rejection tool (ART).^48^ Functional data were smoothed with a 6 mm^3^ full width at half-maximum (FWHM) Gaussian kernel. The T1-weighted structural images were segmented into gray matter, white matter, and cerebrospinal fluid using tissue probability maps. Functional data were bandpass filtered at 0.008-0.1 Hz to reduce noise effects. The six motion parameters and their first-order derivatives were regressed out, and signals from the white matter and cerebrospinal fluid were collected as part of the anatomical component-based noise correction (aCompCor),^49^ along with other confounding factors from the resting state. Regions exhibiting significant intergroup differences in the structural analysis were subsequently selected as seeds for further analysis. Additionally, the hippocampal subfields extracted with FreeSurfer were imported into the CONN toolbox for seed-to-voxel analysis. In the first-level analysis, the average BOLD time series of all regions of interest (ROIs) were extracted, and the correlation coefficients between the BOLD time series of each ROI and brain voxel were computed. For statistical analysis, these correlation coefficients were z-transformed using the Fisher transformation. The z-values for all ROIs were compared between the two groups using analysis of variance (ANOVA) at the second level, the distribution of groups and covariances were added to the General Linear Model (GLM). Comparative analyses between groups and regression analyses were subsequently conducted within the toolbox. Finally, p < 0.001 at the voxel level and family-wise error (FWE) adjusted p < 0.05 at the cluster level.

#### Quality check

All participants' MRI images were reviewed by two researchers to ensure they were of sufficient quality, and it was determined that both the anatomical and functional MRI images of all participants were adequate for preprocessing. Following the preprocessing stage, another review was conducted. During this phase, the segmentation of T1-weighted anatomical images was assessed, including parameters for gray matter segmentation, white matter segmentation, and cerebrospinal fluid segmentation. The normalization of functional and anatomical images to the MNI standard template was also visually checked. Additionally, for the functional images, parameters such as the number of valid and invalid scans, maximum and average motion, and maximum and average global signal changes were evaluated. If a subject had 25% or more invalid scans of the total number of scans and exhibited extreme values in either maximum and average motion or maximum and average global signal changes, they were excluded from the study. Extreme values were defined as those exceeding the threshold of Q3 + 3 IQR (or falling below Q1 - 3 IQR in cases where extremely low values indicated data problems). Here, Q1 and Q3 represent the first and third quartiles of the measure’s distribution across the entire dataset, respectively, and IQR is the interquartile range, which is the difference between Q3 and Q1.^50^

### Quantification and statistical analysis

#### Statistical analysis

Simple descriptive statistics and cognitive scores were analyzed using Jamovi software (version 2.3.19.0). The Shapiro-Wilk test was used to check the normality of the variables. Continuous variables are presented as mean ± standard deviation (mean ± SD) and median (IQR) and categorical variables as frequency (n) and percentage (%). The Independent T-Test and Mann-Whitney U tests were used to analyze MOCA differences between the groups. A two-sided p-value ≤ 0.05 was interpreted as statistically significant.
