# Supplementary material for: Cognitive impairment in tension-type headache is associated with altered hippocampal functional connectivity
Source: iScience. 2025 Oct 23;28(11):113850. doi: 10.1016/j.isci.2025.113850 (PMC12637277; doi:10.1016/j.isci.2025.113850)
Supplement: Document S1. Table S1 [file mmc1.pdf]

iScience, Volume *28*

Supplemental information

Cognitive impairment in tension-type headache is associated with altered hippocampal functional connectivity

Burak Yulug, Ali Yalcınkaya, Shair Shah Safa, Ayse Karakus, Dila Sayman, Seyda Cankaya, Ceyhun Sayman, Ece Ozdemir Oktem, Behçet Ayyildiz, Sevilay Ayyildiz, Uğur Aylak, Bernis Sutcubası, Ramazan Karaca, Mehmet Ozansoy, Umutcan Duran, Halil Aziz Velioglu, Lutfu Hanoglu, and Adil Mardinoglu

Supplementary Table 1: Structural differences between the two groups.

**Region**	**Mean Difference**	**t**	**p-value**	**Cohen's d**
Control > TTH				
**Left Entorhinal Volume**	-279	-3.33	**0.001**	-0.887
**Right Cuneus Area**	-97.9	-2.09	**0.04**	-0.557
**Left Hippocampal Fissure Volume**	-17.4	-2.62	**0.011**	-0.699
**Right Subiculum Body Volume**	-16.7	-2.02	**0.047**	-0.538
**Left Entorhinal Area**	-71.3	-3.41	**0.001**	-0.910
Left Hippocampal Tail Volume	-6.19	-0.292	0.771	-0.07
Left Subiculum Body Volume	-6.49	-0.703	0.484	-0.187
Left CA1 Body Volume	-2.67	-0.476	0.636	-0.127
Left Subiculum Head Volume	-5.21	-0.738	0.463	-0.197
Left Presubiculum Head Volume	0.434	0.106	0.916	0.028
Left CA1 Head Volume	-2.16	-0.146	0.885	-0.038
Left Presubiculum Body Volume	-5.31	-0.799	0.427	-0.213
Left Parasubiculum Volume	1.05	0.311	0.756	0.083
Left Molecular Layer Body Volume	-5.93	-0.859	0.394	-0.229
Left CA3 Body Volume	0.208	0.057	0.954	0.015
Left CA4 Head Volume	-0.980	-0.228	0.820	-0.06
Left CA4 Body Volume	-3.60	-0.950	0.345	-0.253
Left Fimbria Volume	4.89	0.936	0.353	0.249
Left CA3 Head Volume	3.70	0.747	0.458	0.199
Left Hippocampal Body Volume	-22.3	-0.603	0.549	-0.161
Left Hippocampal Head Volume	-2.11	-0.044	0.964	-0.011

Left Whole Hippocampus Volume	-30.6	-0.344	0.732	-0.091
Right Hippocampal Tail Volume	-19.4	-0.937	0.352	-0.250
Right CA1 Body Volume	-4.70	-0.907	0.368	-0.242
Fight Subiculum Head Volume	-7.62	-1.20	0.236	-0.319
Right Hippocampal Fissure Volume	-9.77	-1.44	0.156	-0.383
Right Presubiculum Head Volume	0.355	-0.091	0.927	-0.024
Right CA1 Head Volume	-6.36	-0.406	0.686	-0.108
Right Presubiculum Body Volume	-11.3	-1.64	0.105	-0.438
Right Parasubiculum Volume	-0.988	-0.332	0.741	-0.088
Right Molecular Layer Body Volume	-12.1	-1.80	0.076	-0.481
Right CA3 Body Volume	-2.46	-0.678	0.50	-0.181
Right CA4 Head Volume	-2.65	-0.648	0.519	-0.173
Right CA4 Body Volume	-6.38	-1.74	0.086	-0.464
Right Fimbria Volume	2.14	0.587	0.559	0.157
Right CA3 Head Volume	0.403	0.091	0.929	0.024
Right Hippocampal Body Volume	-59.4	-1.79	0.079	-0.476
Right Hippocampal Head Volume	-24.9	-0.544	0.588	-0.145
Right Whole Hippocampus Volume	-104	-1.21	0.23	-0.323

*In the TTH group compared to the control group, the left entorhinal volume and area, right cuneus area, left hippocampal fissure volume, and right subiculum body volume increased compared to control group. (Abbreviations: TTH: Tension Type Headache)
